# Ten years of morphodynamic data at a micro-tidal urban beach: Cala Millor (Western Mediterranean Sea)

**DOI:** 10.1038/s41597-023-02210-2

**Published:** 2023-05-19

**Authors:** Angels Fernández-Mora, Francisco Fabian Criado-Sudau, Lluís Gómez-Pujol, Joaquín Tintoré, Alejandro Orfila

**Affiliations:** 1 Balearic Islands Coastal Observing and Forecasting System (SOCIB), Palma, Spain; 2 Earth Sciences Research Group, Department of Biology, University of Balearic Islands (UIB), Palma, Spain; 3 Mediterranean Institute for Advanced Studies, IMEDEA (CSIC-UIB), Esporles, Spain

**Keywords:** Physical oceanography, Ocean sciences

## Abstract

Systematic and sustained high quality measurements of nearshore waves and beach morphology are crucial to understand morphodynamic processes that determine beach evolution, to unravel the effects of global warming on sandy coasts and thus improve forecasting models. In 2011 a comprehensive beach monitoring program, the first in the Mediterranean Sea, started at Cala Millor Beach on the island of Mallorca (Spain). The aim was to provide long-term datasets of near-shore morphodynamics in a carbonate sandy micro-tidal and semi-embayed beach fronted by a *Posidonia oceanica* seagrass meadow. We present our morphological and hydrodynamical dataset of Cala Millor covering more than a decade. The dataset includes topobathymetries, shoreline positions obtained from video cameras, meteorological parameters from a weather station, currents, as well as waves and sea level from ADCP measurements and sediment size. This free and unrestricted archived dataset can be used to support the modelling of erosion-deposition patterns, calibrate beach evolution models, and as a result to propose adaptation and mitigation actions under different global change scenarios.

## Background & Summary

Observational data of the near-shore zone are essential to understand key processes related to beach morphodynamics and evolution trends at different scales. They are also key to enhancing the predictive skills of coastal evolution models. Since sandy shorelines are constantly experiencing the effects of wave and nearshore currents, near-shore human assets such as boulevards, infrastructures and buildings in their vicinity are also under threat^1^. This is even more relevant in a climate change scenario where the sea-level rise is expected to impact sandy shorelines during the second-half of the 21st century^2,3^.

Obtaining continuous data of beaches is complex and expensive, due to its variability and the energetic nature of the processes that take place within it (e.g. wave breaking, currents). Historically, the acquisition of datasets of sandy shorelines has been laborious and costly, mainly carried out by land and sea surveys, the analysis of aerial or building based stations images, and more recently of satellite images (for dealing with global scale shoreline datasets^4^). Although there are several short-term or project-based observations capturing nearshore morphodynamics^5–9^, there are few long-term continuous coastal monitoring systems around the globe, particularly regarding full open-access datasets^10–13^.

The continuous and systematic beach monitoring program at Cala Millor beach (CLM) (Mallorca Island, Western Mediterranean Sea) was launched in 2011 as a key project of the Beach Monitoring Facility of the Balearic Islands Coastal Observing and Forecasting System (SOCIB)^14^. This provides high-resolution and sustained near-shore data thus contributing to coastal morphodynamics research.

Our Modular Beach Integral Monitoring System (MOBIMS) comprises: (i) biannual bathymetry and topography surveys, (ii) a 5-camera monitoring system for extracting bimonthly shorelines, (iii) hourly wave and water depth measurements, (iv) real time meteorological measurements, and (v) annual sediment sampling and grain size analysis. Several research studies have been developed from this dataset, including near-shore morphodynamics, beach response to storm events, sea-level rise, rip-currents dynamics and early-warning systems, sea-grass berm formation and dismantling events^15–18^. Coinciding with the 10 years of continuous operations at CLM, and taking advantage of the increasing capacities on data sharing, the full dataset of morphodynamic observations has been released via open access. The current dataset covers beach evolution and hydrodynamic forcing over more than ten years, including mild and energetic conditions as well as erosion/accretion periods at different spatio-temporal scales. This has thus resulted in a comprehensive dataset for near-shore morphodynamics research.

## Methods

### Study site

**Fig. 1:**
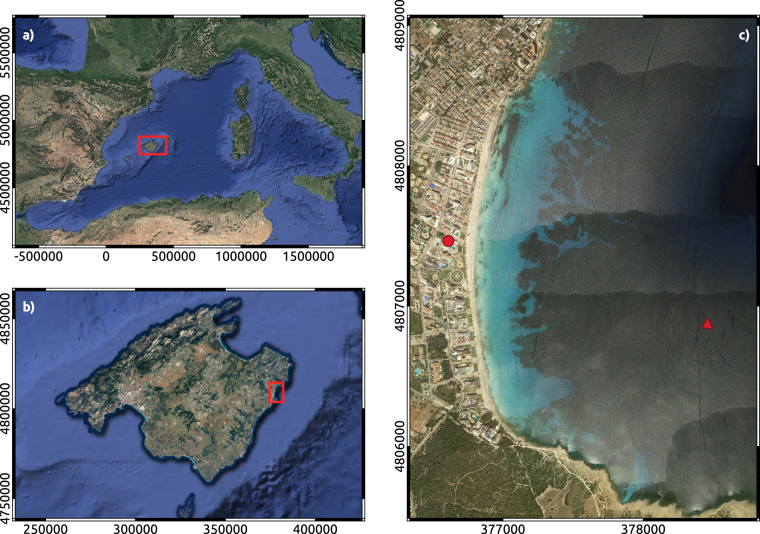
(**a**) Western Mediterranean Sea. Red box indicates the location of Mallorca. (**b**) Mallorca. Red box indicates the location of Cala Millor Beach (CLM). (**c**) Cala Millor beach (CLM). The red dot indicates the location of the video-monitoring station and the red triangle the location of the submerged ADCP.

Cala Millor beach (CLM) is located on the north-eastern coast of Mallorca in the Western Mediterranean Sea (Fig. 1). It is a carbonate sandy beach, ~2 km in length, with a concave shape fronted by a boulevard wall over which hotels and residential houses span over a Holocene dune system and the remnants of a man-filled humid zone^19^. CLM is an intermediate beach with a configuration of transverse and crescentic bars^20^ which means that there is a large alongshore shoreline variability and also rip channels^15,21^.

The beach bottom is characterized by the cropping out of rock reefs, and at depths from 6 to 35 m there are paleochannels and the seagrass meadow of the endemic *Posidonia oceanica*, which acts as a cover to sediment exchange and a friction obstacle to waves^22^. The beach sediments at CLM consist of medium carbonate bioclastic marine sands with a median diameter of approximately 1.8Φ. These sediments correspond to a mid-Holocene attached regressive barrier that prograded landward through a foredune and a field of parabolic dunes.

CLM is exposed to mild-moderate wave conditions, with a mean significant wave height *H*_*s*_ = 0.52 m and peak period *T*_*p*_ = 6.1 s. The wave climate is strongly seasonal dominated, characterized by low and short mostly locally-formed waves due to summer sea breezes, and higher and longer well-developed waves during winter that can reach 4 m in height^18^. Tides are negligible, since the daily tidal range is ~0.2 m. Surge components induced by wind or atmospheric pressure can increase the sea level by up to 1 m^23^. However, there is an inter-annual sea-level variation up to 0.5 m due to inter-annual fluctuations of sea-temperature, internal oscillations in the Mediterranean basin, and the interaction of internal currents in the Western Mediterranean sea^24^.

### Topography surveys

Since 2011, regular and systematic topographic surveys have been conducted at CLM. Two regular field campaigns are scheduled each year: one during early summer, to capture the morphodynamics related to the winter storm exposure (winter profile), and a second during middle autumn, to capture the morphodynamics related to the general mild wave conditions (summer profile).

**Fig. 2:**
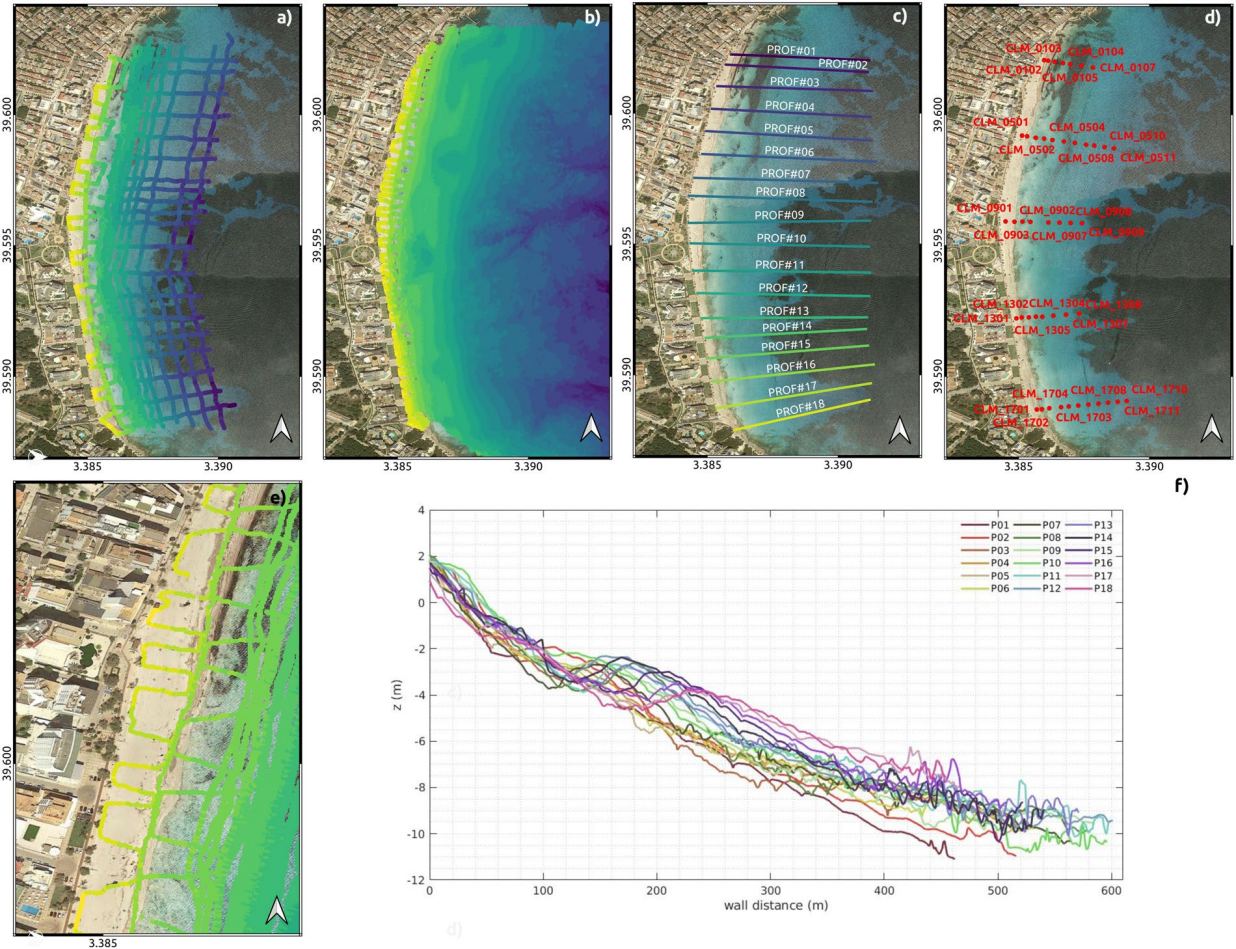
(**a**) Paths followed for bathymetric survey using Biosonic© single-beam echo-sounder in June 2013. (**b**) Bathymetry in June 2019 obtained using a Hypack© multi-beam echo-sounder. (**c**) Location and extension of beach profiles measured during the winter field campaigns. (**d**) Location of the sediment samples. (**e**) RTK-GPS U-path survey example. (**f**) Beach profiles at CLM in March 2022. The location of each profile is shown in panel c).

A 6 m LOA pneumatic boat equipped with an acoustic sonar and GNSS antenna covers the shoreface from approximately 1 m to 15 m water depth. From 2011 to 2015, bathymetries were measured with a BioSonics©single-beam echo-sounder following the paths shown in Fig. 2a. Since 2015, bathymetries have been measured with a Hypack©multi-beam echo-sounder covering the beach extent shown in Fig. 2b.

An RTK-GPS (Real-Time Kinematics) system mounted in a backpack is used to measure sand levels in the surf-zone and swash zone areas. These areas are sufficiently shallow that they are not reached by the echo-sounding system. The system also monitors the emerging beach (dry beach). The shallow waters and dry beach topography covers from approximately 1.4 m depth (to ensure overlapping of the RTK-GPS surveys and the echo-sounding paths) to the upper edge of the dry beach (boulevard wall foot). The RTK-GPS surveys consist of U-paths series covering this range with an average distance between paths of 10–15 m. Figure 2e shows an example of the RTK-GPS U paths.

All points are measured in the European Terrestrial Reference System 1989 (ETRS89), with the 2008 European Gravitational Model adapted to the Spanish Network of High Precision Leveling (EGM08-IGN) as vertical reference datum.

From 2011 to 2018, summer field campaigns covered the full extent of the beach (Fig. 2b), while during the winter field campaigns, only profile measurements were undertaken (Fig. 2c). Since autumn 2018, complete bathymetries have been performed during all field campaigns.

The raw 3D point clouds are post-processed to remove outlier measurements and echoes from the echo-sounding by means of the Hypack©SWEEP software. RTK-GPS measurements are also included during the post-processing to obtain high resolution 3D point clouds of sand-levels along the embayment. Point clouds are computed into 1 × 1 m Digital Elevation Models (DEMs), with the sand level taken as the average elevation within a 1 m radius around any grid point. Profiles and gridded bathymetries are interpolated from the post-processed 3D point clouds.

Profiles are extracted with QGIS software^25^ through a Delaunay triangulation of the point clouds at the transects shown in Fig. 2c and stored in comma-separated value (CSV) files (see Table 1). Cross-shore profiles are provided at a fixed 1 m cross-shore spacing, extending seawards from the boulevard wall foot to ~10 m water depth (Fig. 2f). DEMs are interpolated also from the 3D point cloud in regular rectangular 10 × 10 m equispaced grids. Grid coordinates falling outside of the measurement area are filled with the value −999. Gridded bathymetries are computed in both ETRS89 and WGS84 systems, providing both ASCII DEM files and netcdf files, respectively (see section Data Records).

**Table 1:** Bathymetries and profile dataset - file content (x, y and elevation coordinates of the ETRS89 geodesic system - Lat/Long are referenced in WGS84 ellipsoid).

File type	File name format	File format
Netcdf Gridded bathymetry (WGS84 Lat./Lon.)	clm_YYYYMMDD_bathy.nc	Regular nc variables: Latitude, Longitude, Elevation
ASCII Gridded bathymetry (ETRS89)	clm_YYYYMMDD_bathy_10x10.grd	Row 1 - ncols, number of columns Row 2 - nrows, number of rows Row 3 - XLLCORNER, x coordinate of the lower left corner Row 4 - YLLCORNER, y coordinate of the lower left corner Row 5 - CELLSIZE, grid size Row 6 - NODATAValue, Fill Data Value (set to −999) Row 6 and following rows - z coordinate matrix
Raw 3D point clouds (ETRS89)	raw_clm_YYYYMMDD_bathy(prof).xyz	Column 1 - x coordinate Column 2 - y coordinate Column 3 - z coordinate
CSV Profiles (ETRS89)	clm_YYYYMMDD_prof.csv	Column 1 - Site ID (CLM) Column 2 - Survey date in format ‘yyyymmdd’ Column 3 - Profile ID Column 4 - Chainage (m from origin) Column 5 - Easting (m, x coordinate) Column 6 - Northing (m, y coordinate) Column 7 - Elevation (m, z coordinate)

### Video-Monitoring data and shorelines

Video-monitoring systems are a widely used remote sensing method to track the evolution of beach shorelines, near-shore morphodynamics patterns or beach bathymetry^26–29^. A 5 camera video-monitoring system is used to measure the shoreline position along CLM (Fig. 1). This system is part of the open-source SIRENA video-monitoring system, developed by IMEDEA (Mediterranean Institute of Advanced Studies, CSIC-UIB) in 2007^30^. The video monitoring system, located at 39°59′N and 3°38′E (WGS84) at ~46 m height, captures images at 7.5 Hz starting with the first 10 minutes of each hour of daylight to provide statistical image products (Time Exposure, Snapshots, Variance and TimeStacks). Image products are open-soruce and freely available at https://apps.socib.es/beamon.

**Fig. 3:**
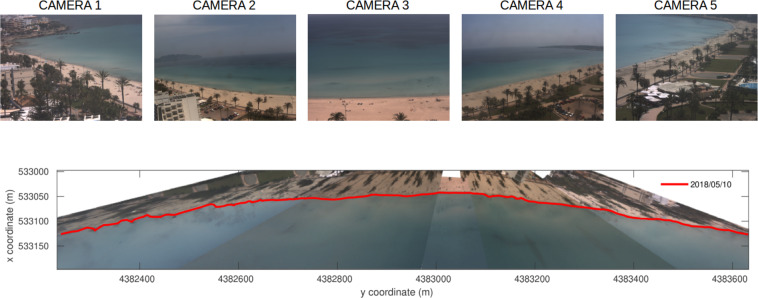
Top: Snapshots from the five cameras of the CLM Sirena video monitoring system. Bottom: Shoreline on May 10th, 2018 obtained by combining exposure images from the video-monitoring system.

Shorelines are digitized biweekly from the georeferenced planview images (ETRS89). Georeferenced planview images from the composition of oblique images are computed using photogeometric techniques (Fig. 3). These techniques account for the rectification of far-field distortions due to lens characteristics (intrinsic calibration), and they link real-world geodetic coordinates and image pixel coordinates (extrinsic calibration). Intrinsic calibration is performed during camera installation by means of the open-source Caltech Camera Calibration Toolbox for Matlab^31^. Extrinsic calibration is obtained using Ground Control Points (GCPs, pixels whose real-world coordinates are known) and the position and orientation (pitch-roll-yaw parameters) of the cameras^32^.

### Waves and water depth

Since 2011, wave characteristics and water depth have been recorded by a Nortek Acoustic Wave and Current (AWAC) profiler deployed at 17 m depth, approximately 1 km offshore from CLM (Fig. 1). This is a combination of a bottom-mounted upward-facing Doppler current profiler (ADCP) and a directional wave gauge. The ADCP measures directional currents along the water column, while directional wave parameters are computed by logging a time-series of pressure, surface velocity and acoustic surface tracking (AST), which estimates the frequency spectrum and other non-directional wave parameters^33,34^. The wave measurement setup uses 1200 samples at 1 Hz starting at the beginning of each hour. Raw data is processed by Nortek QuickWave© software which provides the main wave parameters (non-directional and directional spectrum), surface currents and water depths. Wave dataset spans from 2011/05/20 to the present day, except for a gap from 2019/05/20 to 2019/10/29 due to an instrument failure.

### Real time weather measurements

Weather conditions are measured by a Vaisala weather transmitter (WXT) 520 providing real-time meteorological variables (e.g. atmospheric pressure, temperature, relative humidity, precipitation and wind speed and direction). The station is located close to the video-monitoring station at 49.4 m height.

### Sediment grain size

During summer field campaigns, sediment samples are collected at the same positions covering five profiles along the beach (Fig. 2d). Sediment samples from the dry beach are sampled manually. Submerged beach sediment samples are collected by an Eckman dredge from the pneumatic boat. Sediments are taken to the laboratory, dried, sieved and analyzed with a laser particle size analyser (Malvern Hydro 2000©). Analysis results are post-processed by means of the open-source grain size distribution and statistics package for spreadsheets GRADISTAT^35^ thereby providing the main statistics of grain size (*D*_10_, *D*_50_ and *D*_90_, among other parameters).

Between 2011 and 2018, samples were analyzed with GRADISTAT v. 4.0, and between 2018 and 2020 with v. 8.0. Most of the samples were thinner than 2 mm, and were therefore analyzed exclusively using the Malvern granulometer and processed by the GRADISTAT spreadsheet. For coarser sediment samples, a 100 gr sample was sieved, using 2, 1 and 0.5 mm sieves. These samples were analyzed by integrating the sieving and laser data, obtaining the percentage corresponding to each size before being processed by GRADISTAT.

## Data Records

The full archived dataset can be obtained from SOCIB Data Catalog^36^.

### Topobathymetries datasets

Sand levels are provided in four different formats: (i) raw 3D point-clouds in ASCII files (projected to ETRS89); (ii) the DEMs, provided as ASC Arc/Info ASCII grids (projected to ETRS89); (iii) NetCDF files (projected to WGS84); and (iv) beach profiles (projected to ETRS89), in comma-separated files (csv). Table 1 summarizes the metadata of each type of file available in the repository. Winter campaigns from 2011 to 2018 only provided profile csv files and raw 3D point-cloud files.

### Shorelines

Bi-monthly shoreline positions are provided in ASCII files that contain the x and y coordinates projected to the ETRS89. Shoreline file names follow the expression ‘shl_utm_clm_yyyy_mm_DD_HH_MM.txt’, where ‘shl’ = shoreline, ‘utm’ = universal transverse mercator coordinate system, ‘clm’ = Cala Millor, and ‘yyyy_mm_DD_HH_MM’ stands for the full date (year, month, day, hour, minute) of the corresponding image capture. Video-monitoring images, from which shorelines are extracted, are included together with the corresponding shoreline. Images name follow the expression ‘clm_m_XX_yyyy_mm_DD_HH_MM.png’, where ‘XX’ stands for the camera number, ‘m’ stands for time-exposure image, and ‘yyyy_mm_DD_HH_MM’ for the full date as described above.

### Waves and water depth

Hourly wave and water depth measurements are available in ASCII files with filename format ‘yyyy-mm-dd_mobims-calamillor_scb-awac00X.wap’ (yyyy-mm-dd according to the registration date, with 00X being the ID number of the instrument deployed). Each ASCII ‘∗.wap’ file is accompanied with the corresponding data header file (‘∗.hdr’ extension) that contains all the information related to the instrument deployment, measuring parameters and details of the data contained in the ‘*.wap’ files (Table 2). The wave and water depth dataset spans from 2011/05/20 up to the present day, except for a gap from 2019/05/20 to 2019/10/29 due to an instrument failure.

**Table 2:** Wave and water depth measurements provided as in wave data files.

Column	Variable	Range/Units
1	Month	(1–12)
2	Day	(1–31)
3	Year	
4	Hour	(0–23)
5	Minute	(0–59)
6	Second	(0–59)
7	Spectrum Used	(0-Pressure, 1-Velocity, 3-AST)
8	Significant height (Hm0)	(m)
9	Mean 1/3 height (H3)	(m)
10	Mean 1/10 height (H10)	(m)
11	Maximum height (Hmax)	(m)
12	Mean Height (Hmean)	(m)
13	Mean period (Tm02)	(s)
14	Peak period (Tp)	(s)
15	Mean zero-crossing period (Tz)	(s)
16	Mean 1/3 Period (T3)	(s)
17	Mean 1/10 Period (T10)	(s)
18	Maximum Period (Tmax)	(s)
19	Peak direction (DirTp)	(deg)
20	Directional spread (SprTp	(deg)
21	Mean direction (Mdir)	(deg)
22	Unidirectivity index	
23	Mean Pressure	(dbar)
24	Mean AST Distance	(m)
25	Mean AST Distance (Ice)	(m)
26	No Detects	
27	Bad Detects	
28	Number of Zero-Crossings	
29	Current speed (wave cell)	(m/s)
30	Current direction (wave cell)	(degrees)
31	Error Code	

### Real-time weather data

Real-time weather data are provided as monthly datasets in netcdf format in the CD1.6 convention. Files account for the following variables: air temperature, air pressure, wind speed, average wind from direction, wind gust speed, direction of wind gust, acyclic wind direction (derived), relative humidity, rain accumulation, rain duration, rain intensity, rain peak intensity and voltage. Each variable measurement is accompanied by its corresponding quality control flag (see section Technical Validation).

### Sediment grain size distribution

Sediment grain size is provided in a single spreadsheet (‘BMF_GRAD_Grainsize.xlsx’) containing the main statistical parameters from the laser grain size analysis (Table 3). The first sheet contains the ID code of the samples and their WGS84 Lat/Lon coordinates (Fig. 2d). The rest of the spreadsheets contain the results of the grain-size analysis of all the samples for each single field campaign. Due to the spatio-temporal variability of CLM morphodynamics, some sample records are discontinuous over time, due to water level, wave conditions or rock bed exposure.

**Table 3:** Sediment grain-size statistical variables.

Type of variable	Related variables
Sediment type	Sample Type Textural Group Sediment name
Grain-Size fit Method of Moments (Arithmetic, Geometric, Logarithmic) Folk and Ward Method	Mean Sorting Skewness Kurtosis
Grain Size	*D*_10_, *D*_50_, *D*_90_ among others
Percentage grain type size	

## Technical Validation

### Profiles and bathymetries

Errors in sand elevation are variable and depend on sea temperature, bed smoothness, GNSS platform and wave conditions. Several best-practices and quality control procedures are developed before, during and after surveying as well as during processing to ensure the reliability and quality of the resulting dataset.

#### Echo-sounding and RTK-GPS surveying

Several best-practices and quality control procedures are involved in developing the bathymetries. Regarding the echo-sounding systems: (i) echo-sounding systems are regularly maintained and annually revised by Hypack©technical support; (ii) the water temperature is accounted for by adjusting the echo-sounding frequency and the sailing speed is maintained below 4 knots; (iii) the on-boat GPS system receives real-time differential corrections from the nearest geodesic nodes, and the echo-sounding system is calibrated automatically in relation to the movement of the boat; and (iv) proof sailing patterns are performed: roll (overlapping paths over flat areas in opposite directions), pitch (overlapping path over slopes in opposite directions), heading (partially overlapping paths in the same direction) and point controls (overlapping crossing paths).

In addition, during field campaigns, and if wave conditions permit, control matching points are measured by both the echo-sounding and the RTK-GPS backpack-mounted systems in order to cross-check the reliability of both methods. Gaps in spatial coverage may occur due to shallow sandbars, wave conditions, and mechanical failures.

Similarly, the RTK-GPS software is updated annually and technical maintenance is performed by Leica©technical support. The quality of RTK-GPS measurements is controlled by: (i) a minimum number of accessible satellites (14–16); (ii) an instantaneous and automatic correction from satellite and the closest geodesic nodes (representing a maximum error on x and y coordinates of 0.08 m) and a maximum elevation deviation threshold for 5-time point measurements set to 0.05 m. Mean errors in x and y coordinates are estimated on 0.03 m, and the maximum z coordinate error on 0.05 m.

#### Bathymetries and profiles postprocessing

Echo-sounder measurements are post-processed using Hypack©software. A first automatic filter is applied to prevent spike outliers. Corrections on head, pitch and roll, and heave are automatically applied if required. A human-eye review of the echo-sounding measurements is also performed to remove noise sounding. Once echo-sounding measurements have been processed, RTK-GPS topo-bathymetric measurements are added to the points cloud in order to perform a second review of data and to check elevation matching of the common points between RTK-GPS and the echo-sounder. The full data set is then extracted considering cell points of 1 × 1 m in the post-processed 3D point cloud files (see Table 1).

### Shorelines

Processing cameras images to obtain georeferenced planviews involves intrinsic and extrinsic calibrations. Intrinsic calibration involves the optical correction of images due to lens distortion. After the optical correction, the extrinsic calibration generates georeferenced planviews from images by relating image pixels to real world coordinates. Typically, the resolution ranges between 0.5 and 2 pixels for Cala Millor. Conversely, pixel resolution decreases with distance, but a higher resolution (~0.2 m) is obtained at the shore since cameras are oriented to measure shoreline at the centre of the image^32^. Shoreline positions are digitized from the plan-view images. To prevent differential effects of thermal dilatation of the cameras, shorelines are extracted from images taken at 12:00 AM (CEST).

Repeated RTK-GPS shoreline measurements are taken over the year to validate the accuracy of the shoreline extraction method. The calibration is also repeated if camera movements are detected, ensuring a minimum error in the image georeferencing.

### Wave and water depth validation

The AWAC-AST is replaced during each field campaign, and the corresponding maintenance and calibration are performed to ensure accurate data acquisition for the next deployment. The AWAC-AST data records also account for the corresponding instrument error code. The error code enables users to determine the data reliability. Further details on the definition of AWAC-AST error codes can be found at https://www.nortekgroup.com/.

### Real-Time weather measurements validation

Meteorological stations are controlled daily. Routine maintenance and calibration of instruments ensure the correct functioning and measuring of the instruments. Instrument accuracy information can be found at www.vaisala.com. Quality control procedures for weather measurements are also performed automatically based on the fulfilment of valid ranges (regional and local ranges) of variable values, gradients and spikes and stationary data. The accomplishment of these controls provides each data with a flag determining its reliability (0 - no quality control performed; 1 - good data; 2 - probably good data; 3 - probably bad data; 4 - bad data; 6 - spike; 9 - missing value).

### Sediment grain size

Laser particle size measurements using the Malvern instrument follow the international standards for particle size measurements ISO13320-1^37^. The laser grain-size analyser system is regularly cleaned and maintained. A professional revision, maintenance and calibration of the instrument is also performed annually by the Malvern technical support. During the sample analysis, the system requires verification steps related to optical cleaning and system functioning to ensure the quality of the laser measurements. Each sample analysis is accompanied by a residual value, representing the difference between the measurements and analysis fit. If the residual exceeds a threshold (*R* > 4), the sample analysis is repeated.

## Usage Notes

For ease of use, datasets^36^ are presented in different formats as input for reading software and numerical models.

## Data Availability

The gridded bathymetries (ASCII raster and netCDF files) are interpolated from raw sand elevation data by means of the code file ‘BMF_Gridded_bathymetry.m’ (included in the data repository folder^36^). Code is written in MATLAB (R2018b) and is fully commented. Although MATLAB is a proprietary language, the ‘.m’ files can be read with a text viewer.
